# Two-Step Screening for Depression and Anxiety in Patients with Cancer: A Retrospective Validation Study Using Real-World Data

**DOI:** 10.3390/curroncol31110481

**Published:** 2024-10-23

**Authors:** Bryan Gascon, Joel Elman, Alyssa Macedo, Yvonne Leung, Gary Rodin, Madeline Li

**Affiliations:** 1 MD/PhD Program, Temerty Faculty of Medicine, University of Toronto, Toronto, ON M5S 1A8, Canada; 2 Department of Supportive Care, Princess Margaret Cancer Centre, University Health Network, Toronto, ON M5G 1X6, Canada; joel.elman@mail.utoronto.ca (J.E.); alyssa.macedo@uhn.ca (A.M.); gary.rodin@uhn.ca (G.R.); madeline.li@uhn.ca (M.L.); 3 Department of Psychiatry, Temerty Faculty of Medicine, University of Toronto, Toronto, ON M5S 1A8, Canada; y.leung@northeastern.edu; 4 College of Professional Studies, Northeastern University, Toronto, ON M5X IE2, Canada; 5 Institute of Medical Science, Temerty Faculty of Medicine, University of Toronto, Toronto, ON M5S 1A8, Canada

**Keywords:** psychosocial oncology, distress screening, depression, anxiety, cancer, validation

## Abstract

**Background:** Although screening for distress is recommended by many cancer care guidelines, the uptake of such screening in cancer centers remains limited. Improving the acceptability of screening programs in cancer centers requires a reduction in clinical burden and an improved detection of distress. The purpose of this study was to validate the performance of the two-step screening algorithm used in the Distress Assessment and Response Tool (DART) for identifying cases of anxiety and depression. **Methods**: This retrospective validation study consisted of patients at the Princess Margaret Cancer Centre (PM) who completed the DART, which includes the Edmonton Symptom Assessment System depression (ESAS-D) and anxiety (ESAS-A) items, the Patient Health Questionnaire (PHQ-9), and the Generalized Anxiety Disorder (GAD-7). We evaluated the performance of a two-step screening approach, which modeled the ESAS-D, followed by the PHQ-9 and ESAS-A, then the GAD-7 for predicting a diagnosis of depression and anxiety disorders, respectively. A clinical psychiatric assessment was used as the gold standard reference. **Results:** A total of 172 patients with cancer were included in this study. A total of 59/172 (34%) and 39/172 (23%) were diagnosed with a depression or anxiety disorder, respectively. The sequential administration of the PHQ-9 ≥15 following the ESAS-D (>2) significantly increased the post-test probability of depression from 37% to 60% and improved the performance of predicting depression compared to both the ESAS-D or the PHQ-9 as standalone tests. The sequential administration of the GAD-7 after the ESAS-A did not improve the predictability of an anxiety diagnosis beyond the performance of the ESAS-A or the GAD-7 as standalone tests. **Conclusions:** The present study is among the first to demonstrate that a two-step screening algorithm for depression may improve depression screening in cancer using real-world data. Further research on optimal screening approaches for anxiety in cancer is warranted.

## 1. Introduction

Depression and anxiety disorders are common comorbidities in patients with cancer, with a pooled meta-analytic prevalence of 16.3% and 10.3%, respectively [1]. Given the evidence base for psychosocial interventions improving quality of life, depression, anxiety, and overall health outcomes [2,3], there has been considerable effort over the last decade to improve the identification of patients who may benefit from psychosocial oncology services [4,5,6].

The routine Screening for Distress, the Sixth Vital Sign in cancer care [7], has become an accreditation standard for cancer care organizations globally and has been included in distress management guidelines by the National Comprehensive Cancer Network (NCCN) and other cancer control agencies from as early as 2003 [8,9,10,11]. While studies decades later report significant progress in the uptake of distress screening in cancer centers, a minority of institutions endeavor to screen all patients, with insufficient time to administer screening tools consistently cited as the top barrier to routine screening [4,12].

Improving acceptability and implementation within busy oncology clinics requires that a distress screening tool achieves accurate results while avoiding long surveys in order to maximize screening efficiency and reduce the burden on patients [13]. While ultra-short screening tools may be adequate as a first-stage screen to rule out anxiety and depressive disorders, their poor specificity may lead to the inappropriate referral and use of psychosocial resources [14]. Longer screening tools have improved specificity, although there is no consensus on the optimal psychometric measures [15,16]. Longer measures may also result in clinic flow bottlenecks and be more burdensome to patients, both of which can reduce screening rates [17].

Given the need for more efficient screening approaches and the need to optimize the balance between the sensitivity and specificity of short and long screening tools, a two-step screening approach for distress has been recommended. A two-step screening approach involves the sequential administration of an ultra-short screening tool, followed by a longer screening tool in tandem, such that only patients who screen positive on a highly sensitive ultra-short screening tool go on to complete a longer more specific screening tool to rule in cases of depression or anxiety [18]. In addition to potentially improving the identification of comorbid depression and anxiety disorders in oncology, this approach may also enhance the assessment of distress severity, allowing for a more precise allocation of psychosocial resources and support. This distress screening approach has been recommended in several national cancer anxiety and depression management guidelines (e.g., Canada [19], the USA [20], Australia [21], and Europe [22]), although there is a very limited evidence base supporting which tools and thresholds to use. The few published validation studies have either not been conducted in cancer populations [23,24], used other psychometric measures as a reference gold standard, or evaluated tools not included in the current distress screening guidelines [18,25,26]. Research investigating the validity of a two-step screening approach for depression and anxiety against a gold standard psychiatric assessment in a real-world oncology setting, as recommended in cancer distress management guidelines, is needed.

The Distress Assessment and Response Tool (DART) is a comprehensive distress screening program that was developed and implemented at the Princess Margaret Cancer Centre (PM) in Toronto, Canada, as a routine standard of care since 2010 [27]. The DART is completed by PM patients at every visit, which involves concurrent screening for depression and anxiety using both an ultra-short screening tool, such as single depression and anxiety items from the Edmonton Symptom Assessment System-revised (ESAS-r) [28], and longer screening tools, such as the nine-item Patient Health Questionnaire (PHQ-9) for depression [29,30] and the seven-item Generalized Anxiety Disorder scale (GAD-7) for anxiety [31], consistent with Canadian guidelines [19].

The DART therefore provides a unique opportunity to examine the predictive validity, clinical utility, and workflow impact of a two-step screening approach for depression and anxiety in a real-world oncology setting. Using a clinical psychiatric assessment as the gold standard reference, the primary aim of this study was to examine the screening performance of a two-step screening approach for depression and anxiety in a heterogenous real-world cohort of patients with cancer.

## 2. Materials and Methods

**Study design and population:** This study is a retrospective validation study based on administrative and clinical data routinely collected at PM. The study population consisted of adult patients with a malignant cancer diagnosis at PM in Toronto, Canada, who have completed all measures on the DART and were subsequently referred for psychiatric assessment to the psychosocial oncology (PSO) service at PM between October 2009 and February 2011. This study period was chosen because it preceded the introduction of electronic tailored DART screening in 2012. During the study period, PM sustained a 70% DART screening rate [27], with patients completing all DART measures in full. These data enabled a patient-level combination of ESAS-r, PHQ-9, and GAD-7 scores to model two-step screening approaches across various threshold combinations. PM has a well-developed PSO program, established in 2001, with a multidisciplinary team of social workers, psychiatrists, psychologists, and other health professionals providing diagnosis and treatment for cancer-related psychological distress. All study participants provided informed consent to participate in this study, facilitated by a prompt at the end of the DART, which asks patients to provide consent to their screening data being used for research purposes. All data collection, storage, and analysis procedures were conducted in accordance with the University Health Network (UHN) Research Ethics Board (REB approval #18-5847) in Toronto, Canada.

Data were extracted from patient chart reviews and were linked to DART data. Extracted data from patient charts included any psychiatric disorders diagnosed following DART completion via a comprehensive psychiatric assessment at PSO, type of cancer, disease stage, marital status, gender, date of birth, postal code (used as a socioeconomic status proxy), number of appointments attended one year post-DART, and number of prescriptions for psychoactive medications one year post-DART completion.

Diagnoses of depression and anxiety disorders made by a PSO psychiatrist, following diagnostic criteria outlined in the Diagnostic and Statistical Manual of Mental Disorders fifth edition [32], was used as the gold standard reference for cases of depression and anxiety disorders. A case of depression included one or more of the following diagnoses, as documented at the PSO visit following the patient completion of the DART survey: major depressive disorder, dysthymia, depressive disorder/features secondary to medical or psychiatric condition, and medication-induced depressive disorder. A case of anxiety included one or more of the following diagnoses, as documented at the PSO visit following the patient’s DART completion: generalized anxiety disorder, agoraphobia, panic disorder, and adjustment disorder.

**DART measures:** The DART comprises a combination of validated distress screening tools. These include the ESAS-r, PHQ-9 [29,30], GAD-7 [31], Social Difficulties Inventory (SDI) [33], and Canadian Problem Checklist [34].

The ESAS-r is a screening tool developed and validated for symptom management in palliative and medical oncology [28,35,36]. In the ESAS-r, patients self-report the intensity to which they experience each of nine items pertaining to the physical and mood symptoms that may burden cancer patients on a Likert scale of 0 to 10. The ESAS items include assessments related to pain, tiredness, drowsiness, nausea, appetite, shortness of breath, depression, anxiety, and wellbeing [28].

The PHQ-9 is a validated depression screening tool in oncology [26,29] and contains the following nine items: depression, anhedonia, troubled sleeping, low energy levels, poor eating habits, guilt, troubled concentration, psychomotor abnormalities, and suicidal ideation [30]. Patients report the frequency to which each item is experienced on a four-point scale as follows: not at all (0), several days (1), more than half the days (2), and nearly every day (3). Scores on each item are summed to generate a total PHQ-9 score (0 to 27) from which cutoff scores are applied. Reported cutoff scores in medical populations have ranged from 5 to 10, with sensitivity ranging from 63 to 96 and specificity ranging from 77 to 89, with selective reporting contributing to this wide variability [37].

The GAD-7 scale is a validated screening tool for generalized anxiety disorder in oncology populations [38,39] but has also been used to screen for panic attacks, agoraphobia, social phobias, specific phobias, and posttraumatic stress disorder in other medical populations [40]. Patients report on the frequency to which they experience the following 7 anxiety-related symptoms: nervousness, uncontrollable worry, worrying about too many things, trouble relaxing, restlessness, irritability, and being afraid [31]. Scores on each item are summed to generate a total GAD-7 score (0 to 21) from which cutoff scores are applied. Reported cutoff scores in medical populations for any anxiety disorder have ranged from 5 to 17, with sensitivity ranging from 77 to 91 and specificity ranging from 74 to 83 [40].

**Modeling two-step screening approaches for distress:** Patient-level scores from the ESAS-r, PHQ-9, and GAD-7 surveys were obtained from the DART database. These scores facilitated the examination of performance across different score cutoffs for each measure independently and when combined in a two-step screening approach to identify cases of depression and anxiety.

**Two-step screening for depression:** We modeled a two-step screening approach for depression by defining a positive overall screen as achieving a pre-defined threshold score on both the ESAS depression (ESAS-D) item and the PHQ-9 within the same DART survey. In this screening approach, the single item of the ESAS-D was used as the highly sensitive ultra-short first step of our depression screening algorithm. The ESAS-D item assesses the severity with which patients experience depression, such that “not depressed” is scored as a 0, while “worst possible depression” is scored as a 10. The PHQ-9 was used as the more specific longer 9-item second step in our depression screening algorithm. The impact of varying threshold scores for the ESAS-D and PHQ-9 on post-test probabilities for depression diagnoses was explored.

**Two-step screening for anxiety:** We modeled a two-step screening approach for anxiety in a similar manner to our two-step screening approach for depression. We defined a positive overall screen for anxiety as achieving a pre-defined threshold score on both the ESAS anxiety (ESAS-A) item and the GAD-7 within the same DART survey. The ESAS-A was used as the highly sensitive ultra-short first step in our anxiety screening algorithm. The GAD-7 was used as the more specific longer 7-item second step in our anxiety screening algorithm. The impact of varying threshold scores for the ESAS-A and GAD-7 on post-test probabilities for anxiety diagnoses was explored.

**Statistical analysis:** Descriptive statistics reported the prevalence of anxiety and depression cases in this study population based on a gold standard clinical assessment by PM’s psychosocial oncology service. The sensitivities, specificities, positive predictive values, negative predictive values, and likelihood ratios were calculated for the PHQ-9 and GAD-7 as individual tests across a range of cutoff points. Receiver operating characteristic curve (ROC) analyses were used to compute an area under the curve (AUC), which assessed the ability of the PHQ-9 and GAD-7 to discriminate between cases and non-cases of depression and anxiety, respectively. AUC values range from 0 to 1, with 0.5 indicating a random discriminatory ability and 1 indicating a perfect discriminatory ability. To determine whether the PHQ-9 and GAD-7 scores were associated with clinical care outcomes, Poisson regressions were performed to assess the association between the PHQ-9 and GAD-7 scores and prescribed antidepressant/anxiolytic medications and the number of appointments attended one year post-DART completion.

To explore the most optimal cutoffs for the two-step screening algorithms, post-test probabilities for the two-step models were calculated at multiple cut-points ranging from 1 to 6 on the first steps (ESAS-A and ESAS-D) and at multiple cut-points ranging from 11 to 18 on the second steps (GAD-7 and PHQ-9). These cut-points were used sequentially, such that cases of depression and anxiety were defined as those who scored at or above the threshold on both the first screen (ESAS-A or ESAS-D) and the second screen (GAD-7 or PHQ-9). The formulas used to calculate likelihood ratios and post-test probabilities have been described in greater detail in a previously published validation study, which applied a probabilities approach to validating a two-step screening approach for distress in cancer [18].

To assess the added benefit of the sequential administration of the PHQ-9 and GAD-7 to their respective ESAS items, depression and anxiety outcomes were regressed on summed scores of the ESAS-D vs. the ESAS-D + PHQ-9 and the ESAS-A vs. the ESAS-A + GAD-7, respectively, using logistic regression. Statistical significance was set at *p* < 0.05. Statistical analyses were conducted using SAS 9.2 (SAS Institute Inc., Cary, NC, USA).

## 3. Results

**Characteristics of the study population:** The medical and sociodemographic characteristics of our study population are shown in Table 1. In total, n = 172 patients with cancer were included in this study. The mean age of the cohort was 52 years (standard deviation of 14.4 years) and comprised 58% females. Among the study cohort, n = 39 (23%) patients were diagnosed with an anxiety disorder and n = 59 (34%) patients were diagnosed with a depression disorder via the gold standard psychiatric assessment.

**Figure 1:**
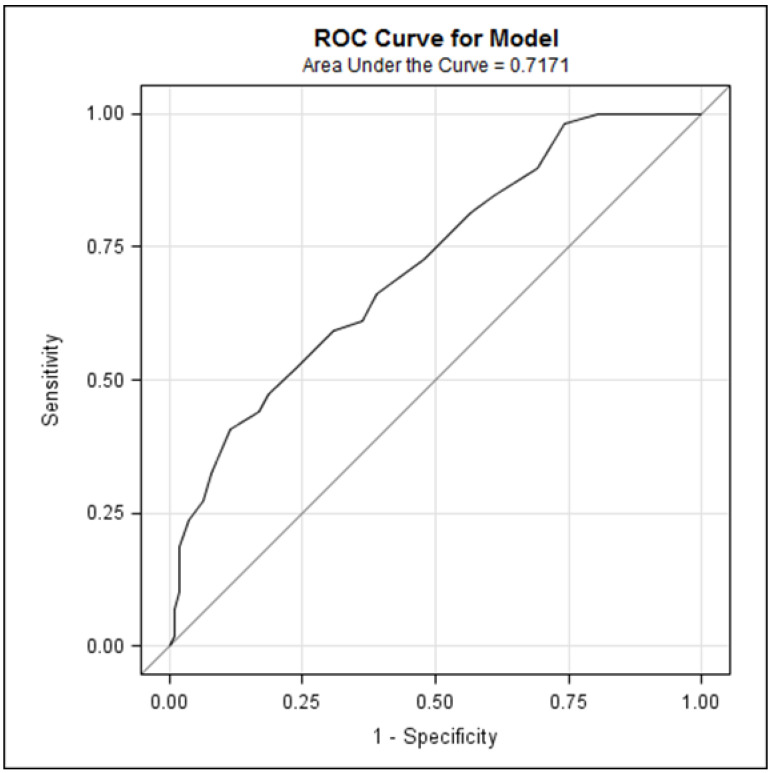
Receiver operating characteristic (ROC) curve of PHQ-9 predicting depression diagnoses.

**Individual screening performance of the PHQ-9 for depression:** Table 2 shows the performance indices of the PHQ-9. Across all PHQ-9 cutoffs, the highest likelihood ratio positive value was 10.53 at both cutoff values ≥ 21 and ≥22, indicating that the likelihood of depression is increased by a factor of 10.53 when scoring at or above these PHQ-9 cutoffs. Statistically significant differences in average PHQ-9 scores were observed between patients with (14.53; SD 6.12) vs. without (9.67; SD 5.45) a depression diagnosis (Table 3; *p* < 0.001). ROC analyses demonstrated that the PHQ-9 achieved an area under the curve (AUC) of 0.72 (Figure 1), indicating an acceptable discriminatory ability for depression cases. Results from the Poisson regression indicated a statistically significant positive association between PHQ-9 summed scores with the number of antidepressants and anxiolytics prescribed (Table 4; *p* < 0.05) and the number of medical appointments attended one year post-DART completion (Table 5; *p* < 0.0001).

**Two-step screening for depression using the ESAS-D and PHQ-9:** Table 6 reports the post-test probability positives and negatives after the sequential administration of the ESAS-D followed by the PHQ-9 for a range of selected cutoffs for both measures. Since two-step screening involves a more sensitive first step and more specific second step, we selected these cutoffs to explore the effect of lowering the cutoff for the first step and raising the cutoff for the second step, relative to commonly published thresholds of each individual measure. At all cutoff combinations examined, post-test probability positives ranged from 49% to 77%, while post-test probability negatives ranged from 7% to 17%. The sequential administration of the PHQ-9 (>15) following the ESAS-D (>2) at the recommended cutoff score [36] increased the post-test probability of a depression diagnosis from 37% to 60% (Table 7). The significance of this improvement was confirmed by the results of the logistic regression (Table 8), which demonstrated that the sequential administration of the ESAS-D followed by the PHQ-9 had significantly improved the performance of predicting a depression diagnosis compared to both the ESAS-D and PHQ-9 as standalone tests (ESAS-D AUC = 0.70 vs. PHQ-9 AUC = 0.72 vs. ESAS-D + PHQ-9 AUC = 0.73).

**Figure 2:**
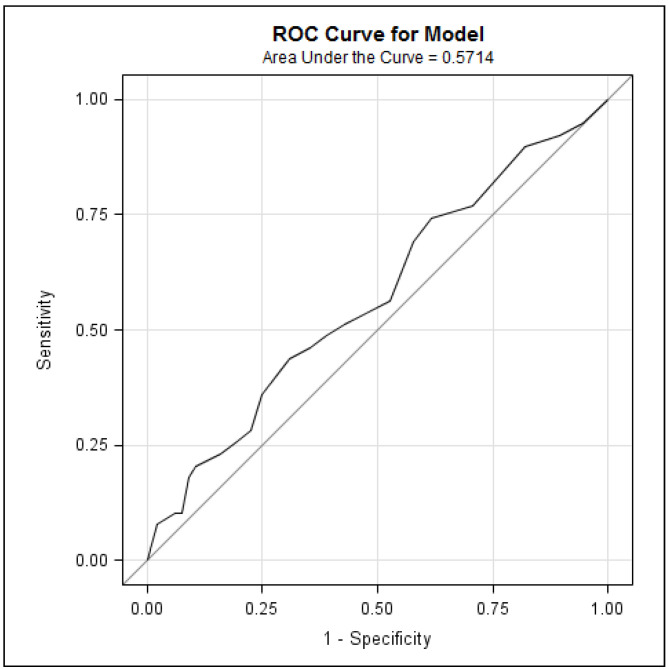
Receiver operating characteristic (ROC) curve of GAD-7 predicting anxiety diagnoses.

**Individual screening performance of the GAD-7 for anxiety:** Table 9 shows the performance indices of the GAD-7. Differences in average GAD-7 scores between patients with vs. without an anxiety diagnosis were not statistically significant (Table 3; *p* = 0.18). ROC analyses indicated that the GAD-7 achieved an AUC of 0.57 (Figure 2), suggesting that the GAD-7 performs slightly better than chance when discriminating between anxiety cases and non-cases. Results from the Poisson regression indicated a statistically significant positive association between GAD-7 scores with the number of dual prescriptions for antidepressants and anxiolytics (Table 4; *p* < 0.0001) and the number of appointments attended one year post-DART completion (Table 5; *p* < 0.0001).

**Two-step screening for anxiety using the ESAS-A and GAD-7:** Table 10 reports the post-test probability positives and negatives after the sequential administration of the ESAS-A followed by the GAD-7 at varying cutoffs on both measures. At all cutoff combinations examined, post-test probability positives ranged from 25% to 39% for the anxiety screen, while post-test probability negatives ranged from 8% to 30%. The sequential administration of the GAD-7 (>15) after the ESAS-A (>2) at the recommended cutoff score [36] increased the post-test probability of an anxiety diagnosis from 24% to 29% (Table 7). Results of the logistic regression (Table 8) confirmed that the sequential administration of the GAD-7 after the ESAS-A did not improve the predictability of an anxiety diagnosis beyond the ESAS-A or the GAD-7 as single screening tests (ESAS-A AUC = 0.55 vs. GAD-7 AUC = 0.57 ESAS-A + GAD-7 = 0.57).

## 4. Discussion

Although two-step emotional distress screening in cancer has been recommended and adopted internationally [19,20,21,22], this approach has not been strongly evidence-based. To our knowledge, the present study is the first to validate a two-step screening algorithm for anxiety and depression against a psychiatric assessment as a gold standard reference using real-world data. Furthermore, the present study demonstrates the application of a probabilities approach [18] to the validation of the PHQ-9, GAD-7, and the two-step screening approaches for depression and anxiety, offering screening performance characteristics under a wide range of cutoff scores to inform contextual implementation efforts that are readily interpretable by clinicians.

The PHQ-9 as a standalone screen had sufficient concordance with a psychiatric assessment for depression, with acceptable sensitivities (0.73–1.00) but poor specificities (0.19–0.52) at commonly used cutoffs (5 to 10). We noted a comparable/slightly reduced performance of the PHQ-9 compared to previous PHQ-9 validation studies in cancer populations [29,41]. This is likely a result of the differences in gold standard reference methods used in other validation studies. Based on post-test probabilities and results from the logistic regression, two-step screening using the ESAS-D followed by the PHQ-9 outperformed both the ESAS-D and PHQ-9 as standalone tests for depression case identification. The present findings demonstrate that using the ESAS-D (>2) and PHQ-9 (>15) sequentially can be used to improve case identification for depression in cancer, which may be accomplished through the implementation of computer-adapted distress screening to enable a tailored patient assessment [27].

The GAD-7 as a standalone screen performed poorly with low sensitivities and specificities at a wide range of cutoffs, as well as AUC values close to 0.5, indicating that its ability to discriminate between anxiety cases and non-cases was insufficient. This screening performance was not improved by two-step screening using the ESAS-A followed by the GAD-7, suggesting that both measures may not adequately capture anxiety diagnoses in patients with cancer in real-world clinical practice. While other validation studies in cancer have found acceptable screening performance of the ESAS-A alone for anxiety when compared against the GAD-7 as a gold standard reference [35], one GAD-7 validation study in cancer using a research interview for GAD as a gold standard reference reported a low sensitivity at the recommended cutoff of >10 (sensitivity 55%), but a more acceptable screening performance at a cutoff of ≥7 (sensitivity of 74% and specificity of 77%) [39]. However, use of the GAD-7 at the same cutoffs for case identification yielded poor PPVs (<8%), potentially due to the relatively low prevalence of GAD in the study’s sample (i.e., ~2%) [39]. Case identification for anxiety diagnoses in cancer is more challenging than for depression due to the more heterogeneous anxiety presentations in cancer. Our study’s finding of relatively poor post-test probabilities of the individual and two-step screening approaches for anxiety, despite the relatively high prevalence of anxiety in our sample (i.e., ~23%), suggests that further studies on alternative approaches to case identification for anxiety in cancer are warranted [38].

*Clinical application:* In busy oncology clinics, a two-step screening approach represents an opportunity to optimize diagnostic accuracy and screening efficiency, as it can maximize the case identification of depression and minimize the burden of questions asked of the patients and as a result, increase acceptability in busy oncology clinics. As with any screening test, cutoffs that provide a desired sensitivity and specificity must be selected by each individual clinic. Limiting anxiety and depression screening to a single item or measure (ESAS-A, ESAS-D, GAD-7, or PHQ-9) would save significant time for patients and be the easiest to implement but would come at the cost of consuming resources for further psychosocial investigations due to tradeoffs between sensitivities and specificities at any one chosen cutoff.

While it may be argued that brief high-sensitivity tools may be sufficient in busy oncology clinics, a more specific tool supports a more effective personalized triage to the right level of psychosocial care. The two-step screening approach optimally increases the efficiency of the screening for distress process. Busy oncology centers with automated computer resources and limited resources for psychiatric investigation may benefit from the implementation of our two-step depression screening approach (ESAS-D ≥2 **→** PHQ-9 ≥15) to improve screening acceptability and screening efficacy. Although the PHQ-9 as a single test demonstrated fair discriminatory power as a standalone tool (AUC = 0.72, Figure 1), its sensitivity is improved with the use of a preceding ultra-short ESAS-D. A valid and sufficient two-step anxiety screening algorithm has yet to be developed.

At PM, through the intelligent programming of the DART, we implemented two-step screening for anxiety and depression [27]. Using an ESAS-D cutoff of two followed by the PHQ-9, approximately 69% of PHQ-9 screens are eliminated from PM cancer clinics (n = 1215) (unpublished data), significantly reducing patient burden. Although using an ESAS-A cutoff of three followed by the GAD-7 also eliminated 67.6% of GAD-7 screens, PM subsequently eliminated GAD-7 from the DART, as it was not found to add any benefit beyond the ESAS-A in our two-step approach to anxiety screening. This single screening approach for anxiety is not consistent with American and European guidelines, which recommend screening with just the GAD-7 [20,22].

For oncology centers where two-step screening approaches may not be possible, screening for depression and anxiety using a single measure may still be of value, with the PHQ-9 and GAD-7 demonstrating better specificity than the ESAS-D and ESAS-A, respectively. Based on the Poisson regression analysis, PHQ-9 summed scores were shown to be predictors of the number of appointments attended one year post-DART implementation and the number of antidepressants, anxiolytics, and both antidepressant/anxiolytics prescribed for any one patient. GAD-7 summed scores were also a predictor of the number of medical appointments attended and anxiolytic prescriptions. These results suggest some value in flagging high summed scores in both measures in terms of patient needs.

*Strengths and limitations:* The main limitation of this study lies in the sample population. The study sample included only oncology patients who (1) have completed the DART and (2) were referred for and accepted psychiatric assessment. This both increases the post-test probability of emotional distress in the study population but also reduces the representativeness of the population, as more marginalized patients tend not to complete distress screening [42]. Furthermore, the staggered implementation and step-wise spread of DART across PM clinics from 2009 to 2011 involved periods in which the DART was systematically not available for completion for some patients [27], further contributing to the relatively limited sample size of this study. Our findings should be interpreted with caution given the relatively small sample size, which may have impacted the precision of our estimates of screening performance.

A strength of this study includes the use of a real-world psychosocial oncology psychiatric assessment as a reference gold standard. Many validation studies of the same measures use other screening tests and/or structured research interviews as a reference gold standard, which may overestimate actual cases of depression and anxiety and inflate performance. Another strength is the use of likelihood ratios and post-test probabilities reported in this study, which are metrics that are more easily interpreted by clinicians to guide screening results for individual patients [18]. Most depression and anxiety screening validation studies report sensitivities and specificities [37,40], which are population measures of validation and have limited application at the individual patient level. Building on the ongoing shift toward adaptive personalized distress screening in cancer, future research should explore the performance of artificial intelligence-based approaches to screening to further enhance diagnostic accuracy, proactive care, and the monitoring of treatment response in PSO [43,44].

## 5. Conclusions

Findings from the present study demonstrated the validity of the ESAS-D (≥2) followed by the PHQ-9 (≥15) as a two-step screening algorithm for depression, which improved diagnostic screening accuracy while minimizing patient and clinician burden compared to the single administration of its constituent screening measures. Further validation studies for anxiety screening approaches in oncology are warranted.

## Figures and Tables

**Table 1:** Study population characteristics.

Characteristic	All Patients (n = 172)
**Age**	
Mean	52
Median	53
Standard deviation (SD)	14.4
Range	19–87
Unknown	n = 5
**Sex**—n (%)	
Male	73 (42%)
Female	99 (58%)
**Marital Status**—n (%)	
Married	89 (52%)
Single	46 (27%)
Divorced	22 (13%)
Unknown	7 (4%)
Widowed	6 (3%)
Common-law	2 (1%)
**Cancer Type**—n (%)	
Breast	32 (19%)
Gastrointestinal	24 (14%)
Hematologic	22 (13%)
Head and neck	21 (12%)
Gynecologic	19 (11%)
Lung	11 (6%)
Brain	8 (5%)
Lymphoma	7 (4%)
Other	28 (17%)
**Anxiety based on psychiatric assessment (reference gold standard)**—n (%)	
Yes	39 (23%)
No	133 (77%)
**Depression based on psychiatric assessment (reference gold standard)**—n (%)	
Yes	59 (34%)
No	113 (66%)

**Table 2:** PHQ-9 performance indices against psychiatric assessment.

PHQ Cut-Off	True Positives	True Negatives	False Positives	False Negatives	Sensitivity	Specificity	PPV	NPV	Likelihood Ratios	
	No. of Correctly Predicted Events	No. of Correctly Predicted Non-Events	No. of Non-Events Predicted as Events	No. of Events Predicted as Non-Events						
									LR Positive	LR Negative
≥0	59	0	113	0	1.00	0.00	0.34	―	1.00	―
≥1	59	3	110	0	1.00	0.03	0.35	1.00	1.03	0.00
≥2	59	5	108	0	1.00	0.04	0.35	1.00	1.05	0.00
≥3	59	10	103	0	1.00	0.09	0.36	1.00	1.10	0.00
≥4	59	12	101	0	1.00	0.11	0.37	1.00	1.12	0.00
≥5	59	22	91	0	1.00	0.19	0.39	1.00	1.24	0.00
≥6	58	29	84	1	0.98	0.26	0.41	0.97	1.32	0.07
≥7	53	35	78	6	0.90	0.31	0.40	0.85	1.30	0.33
≥8	50	44	69	9	0.85	0.39	0.42	0.83	1.39	0.39
≥9	48	49	64	11	0.81	0.43	0.43	0.82	1.44	0.43
≥10	43	59	54	16	0.73	0.52	0.44	0.79	1.53	0.52
≥11	39	69	44	20	0.66	0.61	0.47	0.78	1.70	0.56
≥12	36	72	41	23	0.61	0.64	0.47	0.76	1.68	0.61
≥13	35	78	35	24	0.59	0.69	0.50	0.76	1.92	0.59
≥14	31	86	27	28	0.53	0.76	0.53	0.75	2.20	0.62
≥15	28	92	21	31	0.47	0.81	0.57	0.75	2.55	0.65
≥16	26	94	19	33	0.44	0.83	0.58	0.74	2.62	0.67
≥17	24	100	13	35	0.41	0.88	0.65	0.74	3.54	0.67
≥18	19	104	9	40	0.32	0.92	0.68	0.72	4.04	0.74
≥19	16	106	7	43	0.27	0.94	0.70	0.71	4.38	0.78
≥20	14	109	4	45	0.24	0.96	0.78	0.71	6.70	0.79
≥21	11	111	2	48	0.19	0.98	0.85	0.70	10.53	0.83
≥22	11	111	2	48	0.19	0.98	0.85	0.70	10.53	0.83
≥23	8	111	2	51	0.14	0.98	0.80	0.69	7.66	0.88
≥24	6	111	2	53	0.10	0.98	0.75	0.68	5.75	0.91
≥25	4	112	1	55	0.07	0.99	0.80	0.67	7.66	0.94
≥26	1	112	1	58	0.02	0.99	0.50	0.66	1.92	0.99
≥27	1	112	1	58	0.02	0.99	0.50	0.66	1.92	0.99

Event = diagnosis of depression; PPV = positive predictive value; NPV = negative predictive value; LR = likelihood ratio.

**Table 3:** Comparison of study measures across patients with vs. without depression and anxiety.

	**Diagnosis of Depression**					**Not Diagnosed with Depression**					
**DART Measure**	**Mean**	**Median**	**SD**	**Min**	**Max**	**Mean**	**Median**	**SD**	**Min**	**Max**	***p* Value**
ESAS Depression (ESAS-D)	6.19	7.00	2.78	0	10	4.22	4.00	2.78	0	10	<0.0001
PHQ-9	14.53	14.00	6.12	5	27	9.67	9.00	5.45	0	27	<0.0001
	**Diagnosis of Anxiety**					**Not Diagnosed with Anxiety**					
ESAS Anxiety (ESAS-A)	5.77	6.00	2.90	0	10	5.36	6.00	2.77	0	10	0.39
GAD-7	10.08	9.00	6.25	0	21	8.53	8.00	5.83	0	21	0.18
SD = Standard deviation											

**Table 4:** Poisson regression for association between PHQ-9 and GAD-7 scores and number of prescribed antidepressants and anxiolytics.

Parameter	Prescribed Medication	Estimate	Standard Error	Chi-Square	Pr > Chi-Square
**PHQ-9 Summed Score**	Antidepressant	0.0671	0.0324	4.28	0.0386
	Anxiolytic	0.0891	0.0443	4.06	0.044
	Both	0.1817	0.0454	16.02	<0.0001
**GAD-7 Summed Score**	Antidepressant	−0.00095	0.0327	0	0.9769
	Anxiolytic	0.0817	0.0443	3.4	0.0652
	Both	0.0938	0.0435	4.64	0.0312

**Table 5:** Multivariable Poisson regression for association between PHQ-9 and GAD-7 measures and number of medical appointments attended one year post-DART.

Parameter		DF	Estimate	Standard Error	Wald 95% Confidence Limits Min.	Wald 95% Confidence Limits Max.	Wald Chi-Square	Pr > Chi-Square
**PHQ Summed Score**		1	0.0408	0.0053	0.0303	0.0512	58.46	<0.0001
**Age**		1	−0.0015	0.0027	−0.0067	0.0037	0.33	0.5682
**Sex**	F vs. M	1	0.1054	0.0738	−0.0393	0.25	2.04	0.1533
**Advanced Stage**	No vs. Yes	1	0.7111	0.0915	0.5318	0.8904	60.43	<0.0001
**GAD Summed Score**		1	0.0373	0.0056	0.0264	0.0483	44.47	<0.0001
**Age**		1	−0.0007	0.0027	−0.006	0.0046	0.07	0.7942
**Sex**	F vs. M	1	0.0881	0.0738	−0.0566	0.2327	1.42	0.2328
**Advanced Stage**	No vs. Yes	1	0.7011	0.0915	0.5217	0.8805	58.68	<0.0001

**Table 6:** Effect of varying two-step thresholds for defining cases of depression on post-test probability positives and negatives.

		PHQ-9 SUMMED SCORE															
	Cutoff	≥11		≥12		≥13		≥14		≥15		≥16		≥17		≥18	
		+ve	−ve	+ve	−ve	+ve	−ve	+ve	−ve	+ve	−ve	+ve	−ve	+ve	−ve	+ve	−ve
	≥1	49%	7%	49%	8%	52%	8%	56%	8%	59%	8%	60%	9%	67%	9%	70%	9%
**ESAS Depression**	≥2	50%	10%	50%	11%	53%	11%	57%	11%	60%	12%	61%	12%	68%	12%	71%	13%
	≥3	53%	11%	52%	12%	56%	11%	59%	12%	63%	12%	63%	13%	70%	13%	73%	14%
	≥4	53%	13%	53%	15%	56%	14%	60%	15%	63%	15%	64%	16%	71%	16%	73%	17%
	≥5	58%	12%	58%	13%	61%	12%	64%	13%	68%	13%	68%	14%	74%	14%	77%	15%
	≥6	58%	12%	58%	13%	61%	12%	64%	13%	68%	13%	68%	14%	74%	14%	77%	15%

+ve = post-test probability positive; −ve = post-test probability negative.

**Table 7:** Effect on post-test probabilities of two-step screening at recommended cutoff scores for cases of depression and anxiety.

	Depression			Anxiety		
Performance Index	ESAS-D	PHQ-9	Two-Step	ESAS-A	GAD-7	Two-Step
Cutoff	**≥2**	**≥15**	**≥2 → ≥15**	**≥2**	**≥15**	**≥2 → ≥15**
Sensitivity	0.92	0.47	-	0.92	0.26	-
Specificity	0.21	0.81	-	0.13	0.80	-
Likelihood ratio positive	1.16	2.55	-	1.06	1.31	-
Likelihood ratio negative	0.40	0.65	-	0.60	0.92	-
Post-test probability positive	37%	57%	60%	24%	28%	29%
Post-test probability negative	17%	25%	12%	15%	22%	14%

**Table 8:** Results of logistic regression for screening algorithms predicting clinical diagnosis of depression and anxiety.

**Outcome = Diagnosis of depression**	**Area Under the Curve (AUC)**	**−2 Log likelihood**	**Deviance**	***p* value**
ESAS Depression Score	0.70	202.69	8.87	Top of Form
ESAS Depression Score + PHQ Summed Score	0.73	193.82		0.003
**Outcome = Diagnosis of anxiety**	**AUC**	**−2 Log likelihood**	**Deviance**	***p* value**
ESAS Anxiety Score	0.55	183.50	1.51	Top of Form
ESAS Anxiety Score + GAD Summed Score	0.57	181.98		0.22

**Table 9:** GAD-7 performance indices against psychiatric assessment.

GAD-7 Cutoff	True Positives	True Negatives	False Positives	False Negatives	Sensitivity	Specificity	PPV	NPV	Likelihood Ratios	
	No. of Correctly Predicted Events	No. of Correctly Predicted Non-Events	No. of Non-Events Predicted as Events	No. of Events Predicted as Non-Events					LR Positive	LR Negative
≥0	39	0	133	0	1.00	0.00	0.23	―	1.00	―
≥1	37	7	126	2	0.95	0.05	0.23	0.78	1.00	0.97
≥2	36	14	119	3	0.92	0.11	0.23	0.82	1.03	0.73
≥3	35	24	109	4	0.90	0.18	0.24	0.86	1.10	0.57
≥4	33	30	103	6	0.85	0.23	0.24	0.83	1.09	0.68
≥5	30	39	94	9	0.77	0.29	0.24	0.81	1.09	0.79
≥6	29	51	82	10	0.74	0.38	0.26	0.84	1.21	0.67
≥7	27	56	77	12	0.69	0.42	0.26	0.82	1.20	0.73
≥8	22	63	70	17	0.56	0.47	0.24	0.79	1.07	0.92
≥9	20	76	57	19	0.51	0.57	0.26	0.80	1.20	0.85
≥10	19	81	52	20	0.49	0.61	0.27	0.80	1.25	0.84
≥11	18	86	47	21	0.46	0.65	0.28	0.80	1.31	0.83
≥12	17	92	41	22	0.44	0.69	0.29	0.81	1.41	0.82
≥13	14	100	33	25	0.36	0.75	0.30	0.80	1.45	0.85
≥14	11	103	30	28	0.28	0.77	0.27	0.79	1.25	0.93
≥15	10	107	26	29	0.26	0.80	0.28	0.79	1.31	0.92
≥16	9	112	21	30	0.23	0.84	0.30	0.79	1.46	0.91
≥17	8	119	14	31	0.21	0.89	0.36	0.79	1.95	0.89
≥18	7	121	12	32	0.18	0.91	0.37	0.79	1.99	0.90
≥19	4	123	10	35	0.10	0.92	0.29	0.78	1.36	0.97
≥20	4	125	8	35	0.10	0.94	0.33	0.78	1.71	0.95
≥21	3	130	3	36	0.08	0.98	0.50	0.78	3.41	0.94

Event = diagnosis of anxiety; PPV = positive predictive value; NPV = negative predictive value; LR = likelihood ratio.

**Table 10:** Effect of varying two-step thresholds for defining cases of anxiety on post-test probability positives and negatives.

		GAD-7 Summed Score															
	Cutoff	≥11		≥12		≥13		≥14		≥15		≥16		≥17		≥18	
		+ve	−ve	+ve	−ve	+ve	−ve	+ve	−ve	+ve	−ve	+ve	−ve	+ve	−ve	+ve	−ve
	≥1	29%	8%	31%	8%	31%	8%	28%	9%	29%	9%	32%	9%	38%	8%	39%	8%
**ESAS Anxiety**	≥2	29%	13%	31%	13%	31%	13%	28%	14%	29%	14%	32%	14%	38%	14%	39%	14%
	≥3	26%	28%	28%	27%	28%	28%	25%	30%	26%	30%	28%	30%	35%	29%	35%	29%
	≥4	28%	19%	30%	19%	31%	19%	28%	21%	29%	21%	31%	21%	37%	20%	38%	20%
	≥5	29%	18%	31%	17%	32%	18%	29%	19%	30%	19%	32%	19%	38%	19%	39%	19%
	≥6	29%	18%	31%	17%	32%	18%	29%	19%	30%	19%	32%	19%	38%	19%	39%	19%

+ve = post-test probability positive; −ve = post-test probability negative.

## Data Availability

Data are available by the corresponding author upon reasonable request.
